# Overcoming mechanical adversity in extreme hindleg weapons

**DOI:** 10.1371/journal.pone.0206997

**Published:** 2018-11-07

**Authors:** Devin M. O’Brien, Romain P. Boisseau

**Affiliations:** Division of Biological Sciences, University of Montana, Missoula, Montana, United States of America; Midwestern University, UNITED STATES

## Abstract

The size of sexually selected weapons and their performance in battle are both critical to reproductive success, yet these traits are often in opposition. Bigger weapons make better signals. However, due to the mechanical properties of weapons as lever systems, increases in size may inhibit other metrics of performance as different components of the weapon grow out of proportion with one another. Here, using direct force measurements, we investigated the relationship between weapon size and weapon force production in two hindleg weapon systems, frog-legged beetles (*Sagra femorata*) and leaf-footed cactus bugs (*Narnia femorata*), to test for performance tradeoffs associated with increased weapon size. In male frog-legged beetles, relative force production decreased as weapon size increased. Yet, absolute force production was maintained across weapon sizes. Surprisingly, mechanical advantage was constant across weapon sizes and large weaponed males had disproportionately large leg muscles. In male leaf-footed cactus bugs, on the other hand, there was no relationship between weapon size and force production, likely reflecting the importance of their hindlegs as signals rather than force-producing structures of male-male competition. Overall, our results suggest that when weapon force production is important for reproductive success, large weaponed animals may overcome mechanical challenges by maintaining proportional lever components and investing in (potentially costly) compensatory mechanisms.

## Introduction

**Fig 1:**
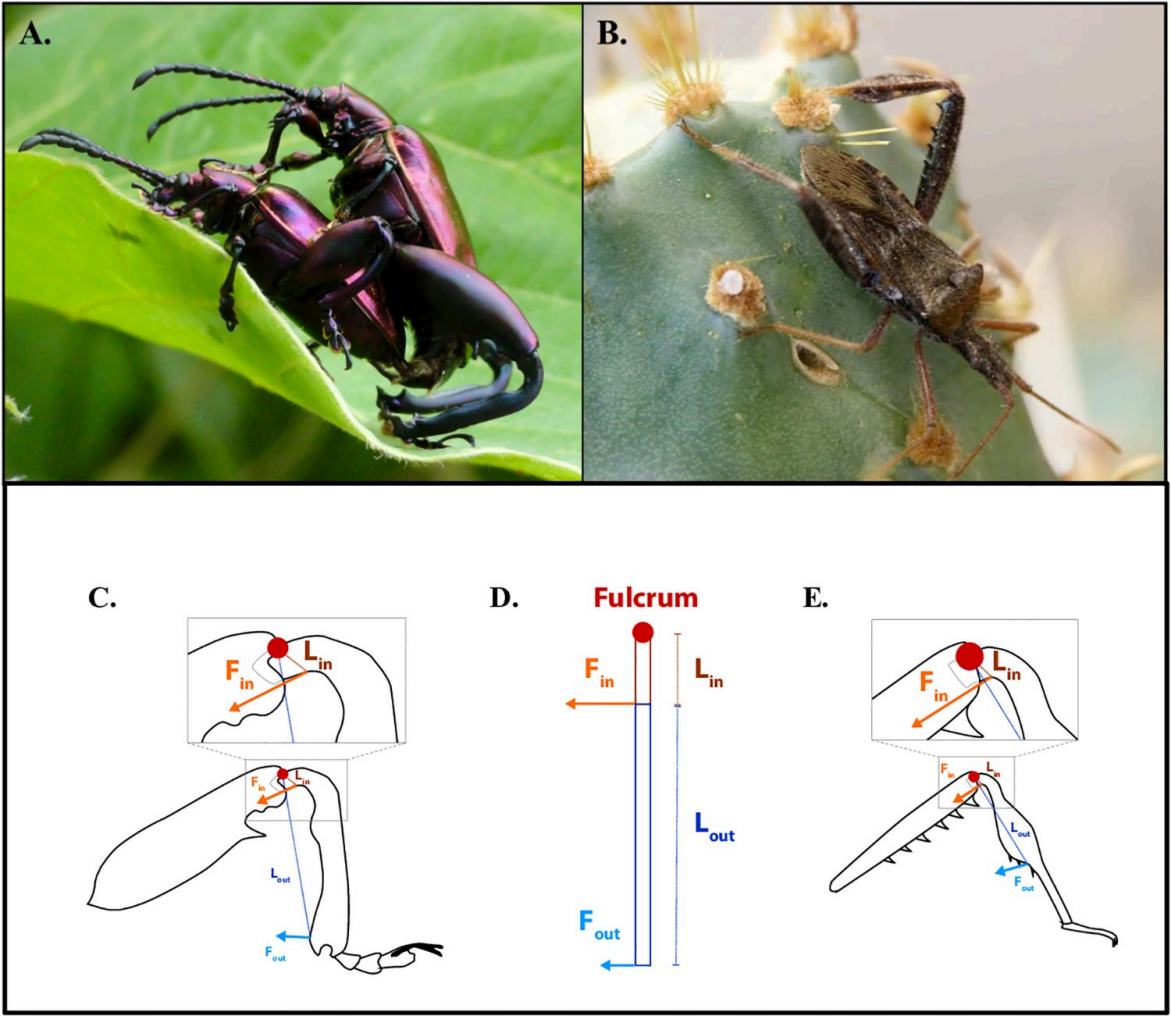
A) Mating *S*. *femorata* (male on top, photo: D. O’Brien). B) Male *N*. *femorata* (photo: R. Boisseau). C-E) Illustrations of lever systems. C) *S*. *femorata* hindlimb. D) Simplified machine. E) *N*. *femorata* hindlimb. Components of lever systems color coded across all structures (L_in_ = input lever (brown), L_out_ = output lever (dark blue), F_in_ = force in (orange), F_out_ = force out (light blue), fulcrum (light red)). All are best described as 3^rd^ order levers.

Animal weapons have a history of strong selection for large size [1–17]. This, in part, results from their role as signals to potential mates [18–20] and rival males [19,21–26] where weapon size functions as an honest signal that captures the genetic and environmental variation underlying individual fitness (hereafter referred to simply as “quality”). Large weapons make the best signals [27,28]. However, as selection pushes weapons toward larger sizes, they face intrinsic, mechanical challenges that impede their performance as fighting tools [19,29–31]. This is because animal weapons, like many other mechanical traits (e.g., jaws of fishes [32–34] or jumping legs in insects [35–37], are lever systems, the components of which must appropriately interact to achieve high performance (e.g., Fig 1C–1E).

All lever systems are composed of a fulcrum (i.e., the pivot about which the lever turns), an “input” lever arm (L_in_), an “output” lever arm (L_out_), an input force (F_in_), and an output force (F_out_). (The relationships between these components are represented by Eq 1).

$$F_{out}=\frac{F_{in}{\;L}_{in}}{L_{out}}$$ (1)

The components of lever systems must remain in proportion to maintain force output (F_out_) [29,30,38]. While increased weapon size may be favored by selection acting toward more efficient signaling or increased reach during combat, variation in the strength of selection and/or constraint experienced by lever components may cause them to scale disproportionally with one another. If, for example, external structures (L_out_—horns, antlers, etc.) are free to become large while internal structures (L_in_ and/or F_in_—tendons, bone, muscle, etc.) are architecturally constrained in their growth, as selection acts to increase overall weapon size, L_out_ may scale with body size at a faster rate than F_in_ and/or L_in_. When this occurs, the mechanical advantage of the lever system will decrease and weapon force output (F_out_) will suffer [19,29,38].

The mechanical limits of lever systems should impede overall trait performance [19,29,30,38]. Large weapons may make compelling signals and even limit the frequency of combat [22,26,39,40]. However, the largest males in a population will still be tested by similarly armed opponent [22,26,39–44]. When this occurs, weapons need to perform well. If not, animals could sustain severe damage and/or death, thereby eliminating their reproductive potential [45,46]. Large traits that function only as signals or deterrents are not sustainable in the context of animal contests. For this reason, animal weapons often represent a selective balance between the need for large, conspicuous signals and strong, force-generating weapons [19,47].

To date, several studies have quantified the relationship between weapon size and mechanical performance [25,29,38,48–58]. Yet, the majority of these studies have focused on one of three ecological/evolutionary scenarios: the claws of crustaceans [49–51,55,57–59], jaws of lizards [25,48,52,60], or weapons that do not function as signals [54,56]. Since the relative importance of signaling and fighting may vary considerably depending on the ecology of the species, further work is necessary to understand how the relationship between weapon size and force production varies across taxa and context and how this variation influences the evolution of sexually selected weapons and signals.

Here, we evaluate weapon performance as a function of weapon size in two species with sexually selected hindleg weapons, frog-legged beetles (*Sagra femorata*: Fig 1A) and leaf-footed cactus bugs (*Narnia femorata*: Fig 1B). We first demonstrate that, for both species, large weapons are essential for competitive success and function as signals of male quality. In this context, we provide the first analysis of fighting success and escalation in the frog-legged beetle. Then, using a strain gauge force-transducer, we measure how weapon force production varies across the natural range of weapon sizes to better understand the balance between selection for increased weapon size and performance. In addition, we measure input lever arm length (L_in_), output lever arm length (L_out_), and muscle mass (estimate of F_in_) in these weapons to evaluate patterns of constraint and compensation involved in maintaining weapon force output. We predicted that large weapons would have relatively (if not absolutely) lower force production than smaller ones (i.e., the “paradox of the weakening combatant” (*sensu* [31]). This would result from decreasing mechanical advantage as weapons become large, which should in turn decrease relative force production (F_out_).

## Materials and methods

### Study species, weapon use, and fighting success

Male frog-legged beetles (Coleoptera, Chrysomelidae, *Sagra femorata*, Dury) have large, sexually dimorphic hindlegs that are used by males in one-on-one battle over direct access to females (Fig 1A). During combat, males attack one another, using their hindlegs to squeeze rival males, pry apart copulating pairs, and steal mates [61,62]. Previous work has demonstrated that males with large weapons have greater reproductive success than smaller weaponed rivals [62]. However, the role weapon size plays in fighting success has never been explicitly tested. Furthermore, it is unclear if the weapons of *S*. *femorata* also function as signals of quality and, by extension, if the paradox of the weakening combatant applies to this species [31]. Below, we analyze 104 naturally observed competitive interactions between male *S*. *femorata* to demonstrate that a) large weapons are essential for competitive success in this species and b) competition escalates in a manner consistent with weapons that also function as signals.

Observations of male-male competition were collected in tandem with measures of mating success reported in [62]. Briefly, a wild population of frog-legged beetles was observed for two breeding seasons. Prior to each season, adult beetles were marked with unique identification numbers. Throughout each season, two researchers scanned the population at regular intervals and recorded all reproductive behavior (including male-male competition). Overall, 104 antagonistic, male-male interactions were observed. Interactions began as males aggressively approached one another, progressed to one of five “escalation levels”, and ended when one contestant either retreated or was forcibly removed from the fighting area. Escalation levels were defined as follows: Level 1) non-violent interaction, Level 2) violent interaction without full combat, Level 3) full combat of mild intensity, Level 4) full combat of high intensity where one contestant retreats, Level 5) full combat of high intensity where one contestant is forcibly removed from the fighting area. Escalation levels were defined prior to analysis. For each interaction, winner, loser, escalation level, and the body (elytra length; EL) and weapon (femur length; FL) size of each contestant were recorded.

Male leaf-footed cactus bugs (Hemiptera, Coreidae, *Narnia femorata*, Stål) also have enlarged, sexually dimorphic hindlegs that are used in male-male competition over reproductive territories (Fig 1B). During fights, rival males back up to one another and use their weapons to squeeze opponents and pull them away from potential mates [26,63–65]. Overall, large weapons offer a competitive advantage over smaller ones. Males with the largest weapons tend to win the most fights and, as a result, have the greatest reproductive success [26,63]. The hindlegs of leaf-footed cactus bugs also function as signals. These weapons are conditionally dependent indicators of male quality [65–68] and male-male interactions escalate more frequently when competitors are similarly matched in weapon size [26], a pattern consistent with classic predictions for weapon-signals [39–44,69]. Similar to frog-legged beetles, the hindlegs of leaf-footed cactus bugs act both as tools of combat and signals of quality. As a result, these weapons are subject to selective conflict between these two functions and, by extension, the paradox or the weakening combatant [31].

### Squeezing force

For analyses of squeezing force, adult *S*. *femorata* were collected from a wild population in Matsuzaka, Mie Prefeture, Japan. Upon capture, measurements of elytra length (body size) and femur length (weapon size [62]) were collected using digital calipers. Animals were housed in 150 ml plastic cups at 25°C and fed Kudzu (*Pueraria spp*.) leaves *ad libitum*. Juvenile leaf-footed cactus bugs were initially collected from a wild population in Gainesville, Florida, USA. Juveniles were shipped to Missoula, Montana, USA where they were housed in 500 mL plastic cups at 28°C and fed cactus fruit and pads (*Opuntia spp*.) *ad libitum*. Measurements of body length (body size) and femur area (weapon size [65]) were collected for each adult using photographs and ImageJ 1.50i software (NIH, USA).

Squeezing force of hindleg weapons was collected using a full bridge, strain gauge force transducer (S1 Fig). The transducer was composed of two needles, which were attached to parallel metal plates. These plates were constructed of flexible brass, which bent as the animal squeezed the needles. Bending of the brass plates (i.e., squeezing force) was recorded using attached strain gauges (model EA-06-062AQ-350, Vishay Measurements Group, NC USA) and was transmitted to a computer (Dell Vosro 220, Dell, TX USA) via amplifier (model 2160 Vishay Measurements Group, NC USA) and AD converter (PowerLab 8sp, ADinstruments, Sydney Australia). Raw values were collected as a change in voltage and converted to a measure of force (N). All measures were recorded in Lab Chart v7.2 (ADinstruments, Sydney AUS).

The relationship between force and measured voltage was identified as non-linear during subsequent analyses, thereby overestimating squeezing force for the largest weapons (particularly large weaponed *S*. *femorata*). The force transducer was therefore calibrated across a range of known weight (2g–100g), a curvilinear ordinary least squares (OLS) regression was fit to the data, and the equation of the best fit curvilinear line (y = 93.362x−10.239x^2^ + 36; F_2,4_ = 1646; *p* < 0.001) was used to correct raw voltage output to accurate force measures. Corrected measures are reported here.

During squeezing trials, animals were held by an observer at the thorax and a single hindleg was placed near the force transducer. For both species, closing force was measured at the most distal point of the true output lever (L_out_). In *S*. *femorata*, L_out_ is equal to the linear distance from the center of the femur-tibia joint (fulcrum) to the distal spine of the tibia (Fig 1C, Figure A in S2 Fig). In *N*. *femorata*, L_out_ is equal to the linear distance from the center of the femur-tibia joint (fulcrum) to the most distal point on the widened “leaf” of the tibia (Fig 1E, Figure B in S2 Fig). Leg placement during squeezing measures aimed to mimic leg position during male-male competition, estimated through personal observation and video recording [D. O’Brien; Miller Lab, University of Florida]. While the animal was squeezing, a second observer annotated each “squeeze”, sorting acceptable “maximum force squeezes” (i.e., performance opportunities, see [70]) from inadequate ones (e.g., poor leg placement on the needles) and removing noise (e.g., insect leg bumping into the needle rather than squeezing it). Even so, due to a lack of cooperation from the animals (especially *N*. *femorata*), there was appreciable variation in leg placement across trials.

Maximum squeezing force was measured across two 2–4 minute trails per insect, during which an average of 8.261 acceptable "maximum force squeezes” were collected. The mean value of all acceptable squeezes for a single animal was calculated as that animal’s overall maximum squeezing force. This measure of maximum squeezing force was used in place of a single, raw measure of maximum squeezing force to better account for variation in animal performance. Insects that held onto the transducer and squeezed constantly throughout either trial were removed from the analysis, ensuring that “squeezing endurance” was not measured in place of maximum squeezing force.

### Dissections (muscle mass and measures of L_in_ and L_out_)

Hindleg muscle mass was collected from a subset of *S*. *femorata* (males: n = 88, females: n = 85) and all *N*. *femorata* used in squeezing analyses. Whole hindlegs (*S*. *femorata*) and femurs (*N*. *femorata*) were dissected, dried at 70°C, and weighed. After initial weighing, muscle was digested by fully submerging the leg in 10% KOH and incubating at 70°C for 12 (*S*. *femorata*) or 8 (*N*. *femorata*) hours to ensure total dissolution of soft tissues [71]. After digestion, hindlimbs were dried at 70°C and weighed a second time. The difference between the first and second weighing was taken as an estimate of dry muscle mass. Muscle mass was taken from a single leg (leg used in squeezing trial when available).

Hindlegs were dissected in a subset of *S*. *femorata* (n = 27) to determine the precise internal structure of the leg and to gain accurate measures of L_in_ and L_out_ (Fig 1C, Figure A in S2 Fig). L_in_ was identified as the linear distance from the center of the femur-tibia joint to the muscle attachment sclerite of the tibia. L_out_ was identified as the linear distance from the center of the femur-tibia joint to the distal spine of the tibia. Measurements of L_in_ and L_out_ were collected using photographs of dissected legs and ImageJ 1.50i software (NIH, USA). From these measures, the relationships between L_out_ and tibia length and L_in_ and tibia length were calculated using ordinary least squares regression [72]. There were no significant sex differences in these relationships (95% CI intercept L_in_ for males [-0.227, 0.972] and females [-0.294, 0.691], 95% CI slope L_in_ for males [-0.29, 0.11] and females [-0.038, 0.135], 95% CI intercept L_out_ for males [-2.144, 3.397] and females [-0.855, 1.11], 95% CI slope L_out_ for males [0.554, 1.197] and females [0.776, 1.086]). Thus, male and female data were combined into the two regressions reported here (L_in_: y = 0.079x + 0.03, F_1,24_ = 91.26, *p* < 0.0001; L_out_: y = 0.903x + 0.39, F_1,24_ = 795.8, *p* < 0.0001). Equations from these regressions were then used to estimate L_in_ and L_out_ for every beetle using measures of tibia length described above.

Similarly, hindlegs of *N*. *femorata* were dissected to identify exact measures of L_in_ and L_out_. L_in_ was identified as the linear distance from the center of the femur-tibia joint to the attachment point of the flexor muscle on the tibia (Fig 1E, Figure B in S2 Fig). L_out_ was identified as the linear distance from the center of the femur-tibia joint to the most distal point on the widened “leaf” of the tibia. Both L_in_ and L_out_ were directly measured in all animals using photographs of dissected legs and ImageJ 1.50i software (NIH, USA).

### Statistical analyses

All statistical analyses were performed in R 3.3.2 (R Core Development Team 2016). To analyze competitive interactions between male *S*. *femorata*, “Male A” was identified as the male approached by a competitor and the approaching competitor was identified as “Male B”. Logistic regression was used to assess interaction outcome (Male A or B wins) in relation to the difference in competitor weapon size (FL_A_ − FL_B_). In addition, a mixed effects model (R package lme4 [73]) was used to determine the role weapon size plays in competitive success while controlling for repeated measures of the same individual across multiple interactions. This model included weapon size as a fixed effect, and interaction number and competitor as random effects. Finally, a generalized linear model (family = “Gaussian”) was used to assess the relationship between escalation level and absolute difference in competitor weapon size (|FL_A_ − FL_B_|).

Ordinary least squares (OLS) regression was used to assess all scaling relationships [72] and all data were log_10_ transformed prior to analysis. For both species and in both sexes, weapon size (*S*. *femorata*, femur length; *N*. *femorata*, femur area), L_in_, L_out_, and muscle mass were regressed on body size (*S*. *femorata*: elytra length, *N*. *femorata*: body length) in separate models.

Maximum squeezing force was regressed on weapon size in both species and both sexes to assess overall weapon force output. For male *S*. *femorata*, linear models with interaction terms between weapon size and muscle mass were constructed to further explore the effect of weapon size, muscle mass, and their interaction on squeezing force. Differences in maximum squeezing force between sexes were calculated using *t*-tests.

To determine whether the observed increase in muscle mass relative to body size represented a compensatory mechanism, 95% confidence intervals were generated from the OLS regression and used to compare the observed scaling relationship between muscle mass and body size to the expected, isometric relationship (*β*_*0*_ = 3 for volumetric measures). If the observed slope was greater than expected (i.e., *β* > 3), it was considered a compensatory mechanism [38].

Finally, since mechanical advantage is expected to decrease in the absence of compensation as weapons grow large [29,38], log_10_ mechanical advantage ([L_in_]/[L_out_]) was regressed against weapon size.

## Results

### Fighting success in *S*. *femorata*

**Fig 2:**
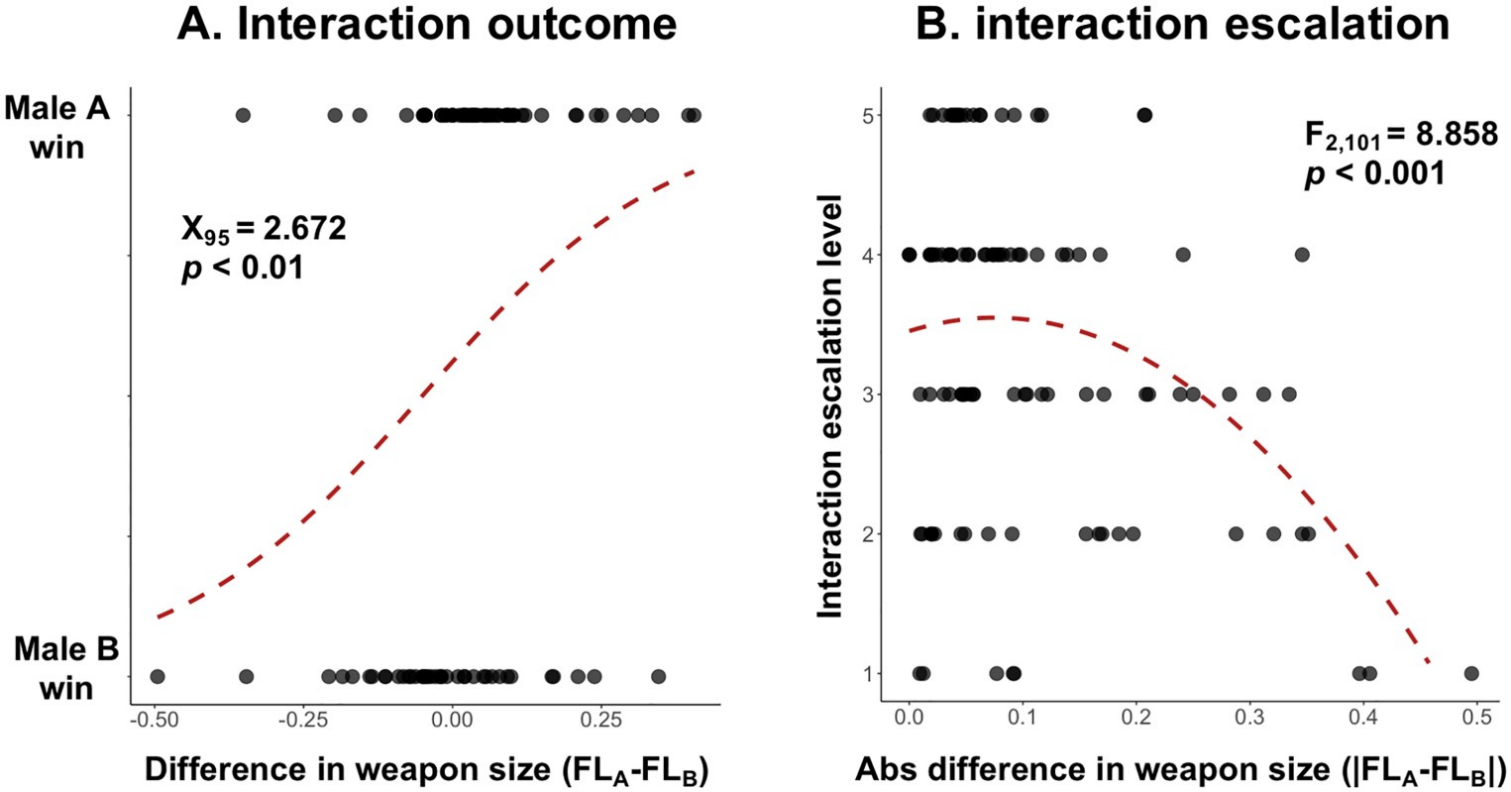
Interaction outcome and escalation in *S*. *femorata*. A) Interaction outcome. Red line represents logistical regression of interaction outcome (Male A or B wins) in relation to the difference in competitor weapon size (Femur length; FL_A_ − FL_B_). Positive x values indicate Male A had a larger weapon size than the opponent. Negative x values indicate that Male B had a larger weapon size than the opponent. B) Interaction escalation. Red line represents generalized linear model between interaction escalation level and absolute difference in weapon size (Femur length; |FL_A_ − FL_B_|).

In *S*. *femorata*, large weaponed males won 69.15% of competitive interactions and this trend held after controlling for repeated measures of contestants across multiple interactions (β = 0.452 ± 0.155, z = 2.92, *p* < 0.01). In addition, the probability that the larger weaponed male won increased as the difference in competitor weapon size increased (z_95_ = 2.672, *p* < 0.01; Fig 2A). Overall, weapon size appears to be a key component of competitive success where large, strong weapons offer an advantage over smaller ones. In addition, these weapons seem to function as competitive signals. Across interactions, escalation level decreased as absolute difference in competitor weapon size increased (F_2,101_ = 8.658, *p* < 0.001; Fig 2B). This is consistent with classic contest theory [39–44,69] and recent empirical evidence [22,26], which predicts distinct patterns of escalation when weapons are also used as signals. Overall, the hindlegs of *S*. *femorata* act as both weapons of male-male battle *and* signals of quality and are therefore subject to conflict between these two functions.

### Squeezing force

**Fig 3:**
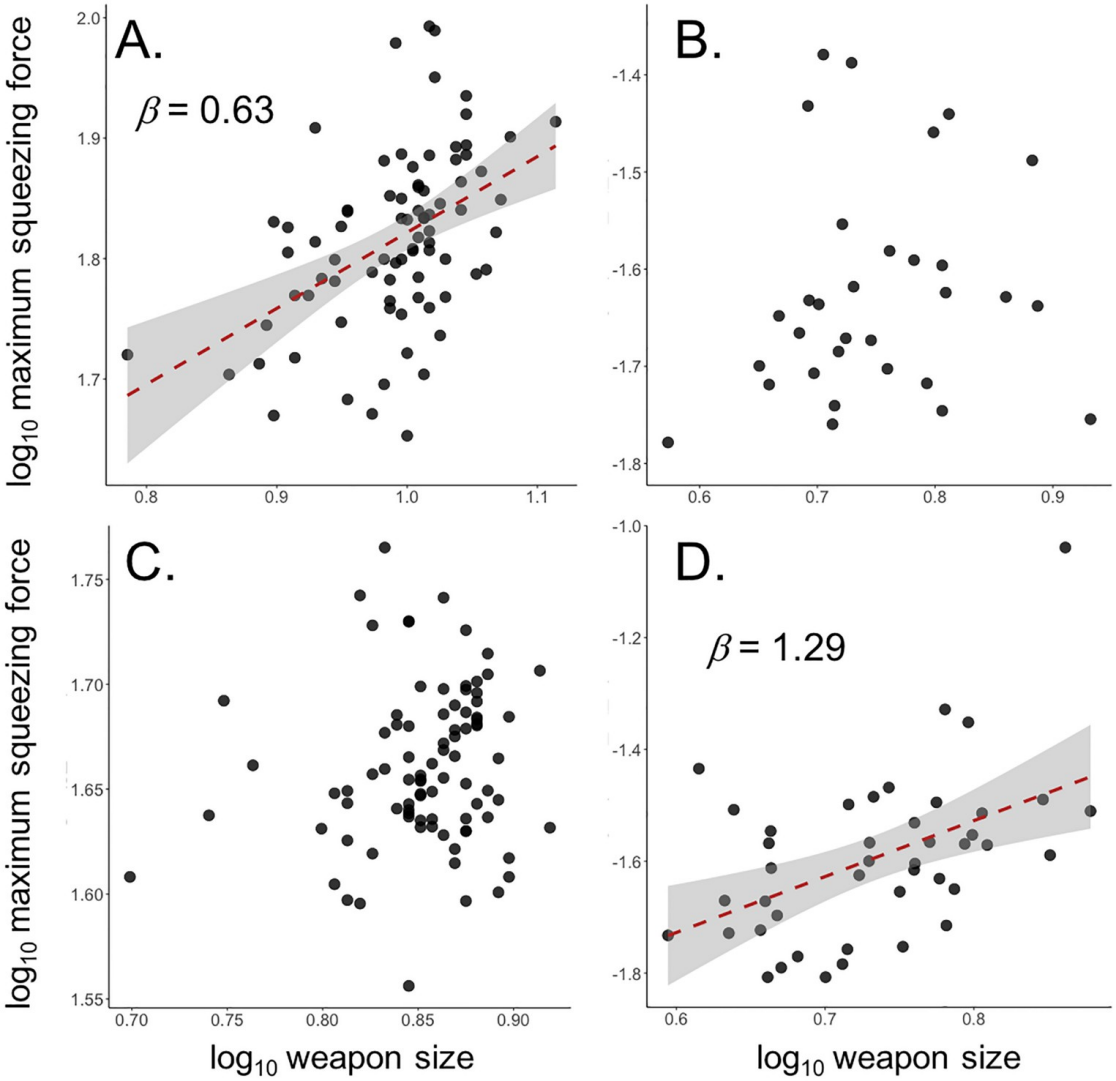
Relationship between maximum squeezing force and weapon size for A) male *S*. *femorata*, B) male *N*. *femorata*, C) female *S*. *femorata*, and D) female *N*. *femorata*. Red lines represent OLS regression. Shaded areas represent 95% confidence intervals around OLS regressions. Lines omitted for non-significant regressions.

In male *S*. *femorata*, maximum squeezing force increased hypoallometrically with weapon size (Fig 3A; Table 1). There was no significant interaction between muscle mass and weapon size on maximum squeezing force (t_84_ = 0.669, *p* = 0.505). In female *S*. *femorata*, there was no significant relationship between maximum squeezing force and weapon size (Fig 3C; Table 1). In *S*. *femorata*, maximum squeezing force was higher in males than in females (mean_male_ = 0.338N; mean_female_ = 0.109N; t_113.42_ = 15.996, *p* < 0.0001).

**Table 1:** Models for squeezing force analyses (y ~ x format). SE = standard error. R^2^ = adjusted R^2^. *S*. *femorata*: weapon size = femur length, body size = elytra length. *N*. *femorata*: weapon size = femur area, body size = body length.

**Weapon size**									
	Model	Intercept	SE	Slope	SE	n	R^2^	F_(df)_	*p* value
*S*. *femorata* (male)	weapon size ~ body size	-0.443	0.048	1.267	0.042	95	0.906	903.6 _1,93_	< 0.0001
*S*. *femorata* (female)	weapon size ~ body size	-0.29	0.063	1.036	0.057	99	0.769	327 _1,97_	< 0.0001
*N*. *femorata* (male)	weapon size ~ body size	-2.143	0.199	2.849	0.1967	39	0.846	209.8 _1,37_	< 0.0001
*N*. *femorata* (female)	weapon size ~ body size	-1.516	0.1491	2.12	0.141	44	0.841	227.6 _1,42_	< 0.0001
**Muscle mass**									
	Model	Intercept	SE	Slope	SE	n	R^2^	F_(df)_	*p* value
*S*. *femorata* (male)	muscle mass ~ body size	-2.912	0.199	3.46	0.176	88	0.819	387.9 _1,86_	< 0.0001
*S*. *femorata* (female)	muscle mass ~ body size	-2.975	0.278	3.278	0.251	85	0.672	170.4 _1,83_	< 0.0001
*N*. *femorata* (male)	muscle mass ~ body size	-0.382	0.756	3.027	0.745	39	0.252	16.51 _1,37_	< 0.001
*N*. *femorata* (female)	muscle mass ~ body size	-0.936	0.322	3.438	0.304	44	0.71	128.2 _1,42_	< 0.0001
**L**_**in**_									
	Model	Intercept	SE	Slope	SE	n	R^2^	F_(df)_	*p* value
*S*. *femorata* (male)	L_in_ ~ body size	-1.316	0.179	1.023	0.159	95	0.301	41.56 _1,93_	< 0.0001
*S*. *femorata* (female)	L_in_ ~ body size	-0.481	0.728	0.166	0.066	100	0.051	6.327 _1,98_	0.014
*N*. *femorata* (male)	L_in_ ~ body size	-1.795	0.246	1.324	0.243	39	0.421	29.66 _1,37_	< 0.0001
*N*. *femorata* (female)	L_in_ ~ body size	-2.036	0.204	1.483	0.192	44	0.576	59.39 _1,42_	< 0.0001
**L**_**out**_									
	Model	Intercept	SE	Slope	SE	n	R^2^	F_(df)_	*p* value
*S*. *femorata* (male)	L_out_~ body size	-0.248	0.178	1.016	0.157	95	0.302	41.7 _1,93_	< 0.001
*S*. *femorata* (female)	L_out_~ body size	0.582	0.722	0.164	0.065	100	0.051	6.318 _1,98_	0.014
*N*. *femorata* (male)	L_out_~ body size	-0.085	0.111	0.719	0.11	39	0.526	43.15 _1,37_	< 0.0001
*N*. *femorata* (female)	L_out_~ body size	-0.469	0.139	1.048	0.131	44	0.596	64.29 _1,42_	< 0.0001
**Mechanical advantage**									
	Model	Intercept	SE	Slope	SE	n	R^2^	F_(df)_	*p* value
*S*. *femorata* (male)	mechanical adv. ~ weapon size	-0.957	0.367	-0.101	0.372	13	-0.087	0.074 _1,11_	0.791
*S*. *femorata* (female)	mechanical adv. ~ weapon size	-0.786	0.329	-0.382	0.444	13	-0.022	0.742 _1,11_	0.408
*N*. *femorata* (male)	mechanical adv. ~ weapon size	-1.268	0.052	0.227	0.07	39	0.206	10.6 _1,36_	0.002
*N*. *femorata* (female)	mechanical adv. ~ weapon size	-1.221	0.068	0.157	0.093	44	0.042	2.855 _1,41_	0.01
**Squeezing force**									
	Model	Intercept	SE	Slope	SE	n	R^2^	F_(df)_	*p* value
*S*. *femorata* (male)	Squeezing force ~ weapon size	1.183	0.134	0.63	0.135	95	0.18	21.68 _1,93_	< 0.001
*S*. *femorata* (female)	Squeezing force ~ weapon size	1.531	0.01	0.153	0.116	100	0.007	1.736 _1,98_	0.191
*N*. *femorata* (male)	Squeezing force ~ weapon size	-1.592	0.306	0.353	0.409	39	-0.007	0.756 _1,36_	0.39
*N*. *femorata* (female)	Squeezing force ~ weapon size	-2.241	0.272	1.289	0.369	44	0.211	12.21 _1,41_	< 0.01

In male *N*. *femorata*, there was no significant relationship between maximum squeezing force and weapon size (Fig 3B; Table 1) [38]. In females, maximum squeezing force increased isometrically with weapon size (Fig 3D; Table 1). There was no significant difference in maximum squeezing force between sexes in *N*. *femorata* (t_96.286_ = -0.0396, *p* = 0.693).

### Morphological measures of lever components

**Fig 4:**
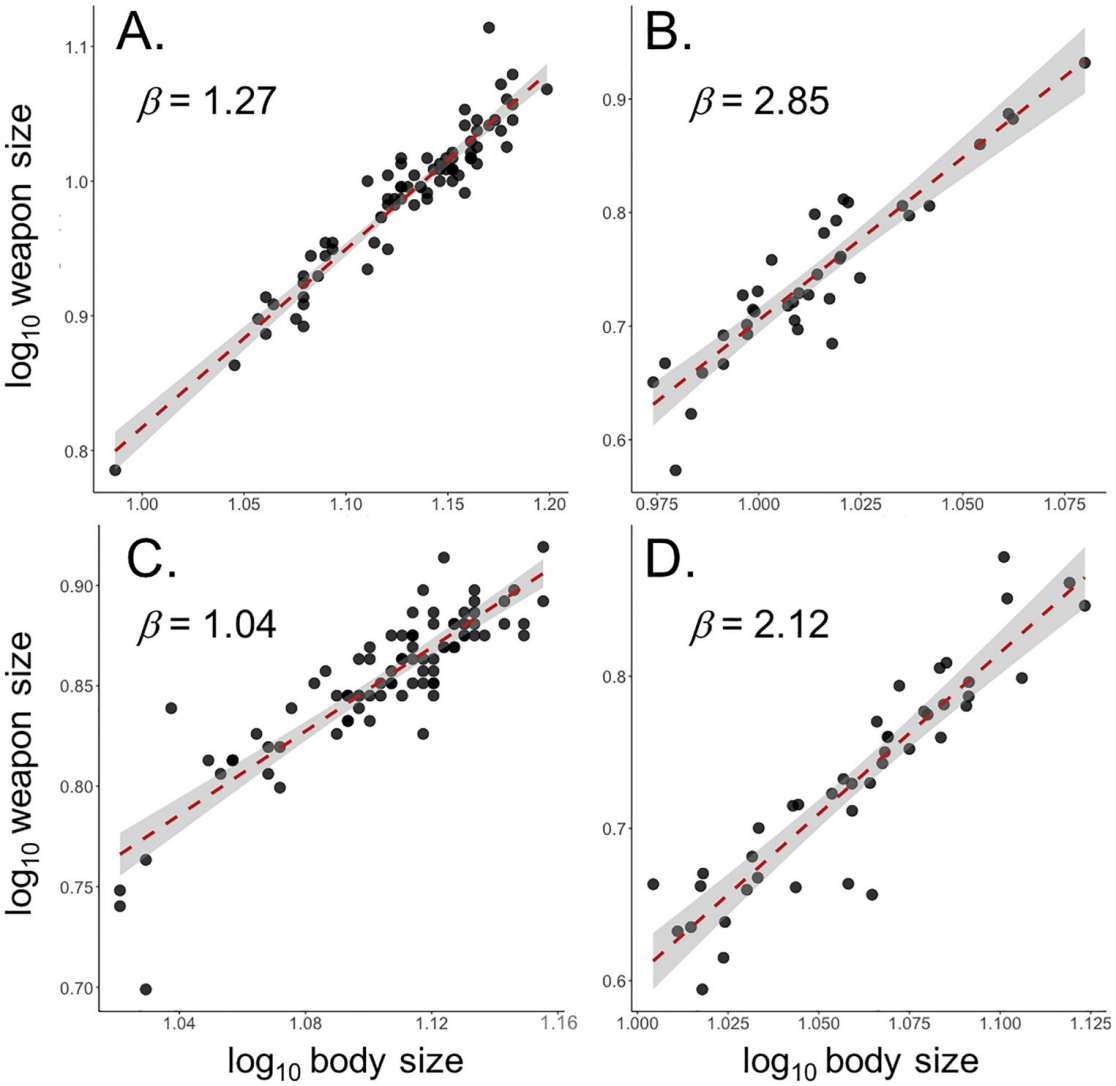
Relationship between weapon size and body size for A) male *S*. *femorata*, B) male *N*. *femorata*, C) female *S*. *femorata*, and D) female *N*. *femorata*. Red lines represent OLS regression. Shaded areas represent 95% confidence intervals around OLS regressions.

**Fig 5:**
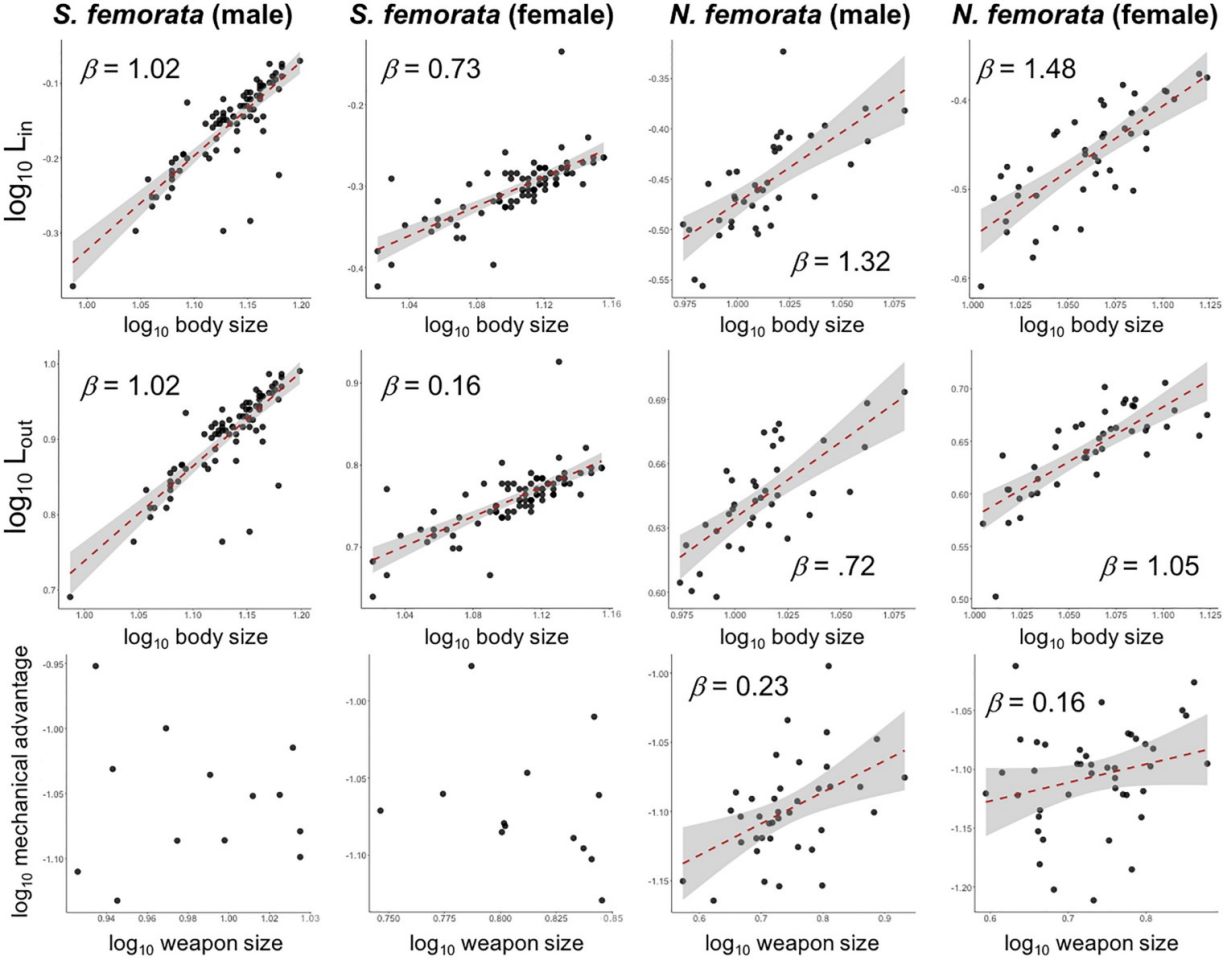
Relationships between lever components/mechanical advantage and body size for A) *S*. *femorata* males, B) *S*. *femorata* females, C) *N*. *femorata* males, and D) *N*. *femorata* females. Top: L_in_ vs. body size. Middle: L_out_ vs. body size. Bottom: mechanical advantage vs. body size. Red lines represent OLS regression. Shaded areas represent 95% confidence intervals around OLS regressions. Lines omitted for non-significant regressions.

A summary of all morphological measures is provided in S1 Table. In *S*. *femorata*, weapon size increased hyperallometrically with body size in males and isometrically in females (Fig 4A and 4C; Table 1). L_in_ increased isometrically with body size in males and hypoallometrically with body size in females (Fig 5; Table 1). L_out_ increased isometrically with body size in males and hypoallometrically with body size in females (Fig 5; Table 1). There was no signifcant relationship between mechanical advantage and weapon size in males or females (Fig 5; Table 1).

In *N*. *femorata*, weapon size increased hyperallometrically with body size in males and isometrically with body size in females (Fig 4B and 4D; Table 1). L_in_ increased increased hyperallometrically with body size in both males and females (Fig 5; Table 1). L_out_ increased hypoallometrically with body size in males and isometrically with body size in females (Fig 5; Table 1). Mechanical advantage increased hypoallometrically with weapon size in both males and females (Fig 5; Table 1).

**Fig 6:**
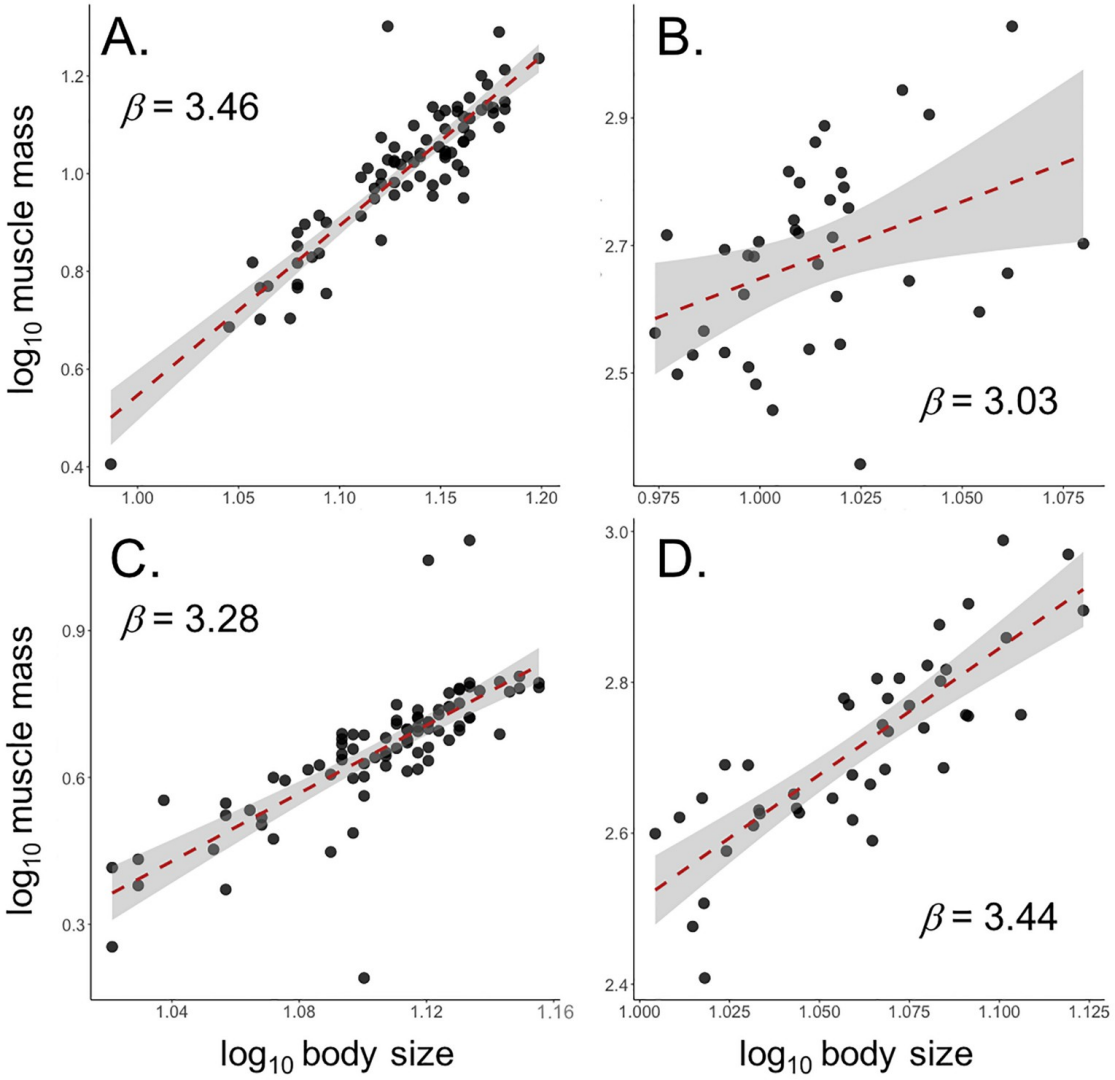
Relationship between hindlimb muscle mass and body size for A) male *S*. *femorata*, B) male *N*. *femorata*, C) female *S*. *femorata*, and D) female *N*. *femorata*. Red lines represent OLS regression. Shaded areas represent 95% confidence intervals around OLS regressions.

In male *S*. *femorata*, muscle mass increased hyperallometrically with body size, which is consistant with a compensatory mechanism (*β* = 3.406 ± 0.176, F_1,86_ = 387.9, *p* < 0.0001; Fig 6A; Table 1). In females, muscle mass also increased hyperallometrically with body size ((*β* = 3.278 ± 0.251, F_1,83_ = 170.4, *p* < 0.0001; Fig 6C; Table 1).

In male *N*. *femorata*, muscle mass scaled isometrically with body size (males: *β* = 3.027 ± 0.745, F_1,37_ = 16.51, *p* < 0.001; Fig 6B; Table 1). Muscle mass scaled hyperallometrically with body size in female *N*. *femorata* (*β* = 3.438 ± 0.304, F_1,42_ = 128.2, *p* < 0.0001; Fig 6D; Table 1).

## Discussion

We measured weapon force output as a function of weapon size in two wild, sexually selected weapon systems, frog-legged beetles (*S*. *femorata*) and leaf-footed cactus bugs (*N*. *femorata*). In frog-legged beetles, weapon force output increased hypoallometrically (*β* = 0.630 ± 0.135) with weapon size, suggesting large weaponed males have relatively lower, but absolutely higher, force production than smaller rivals (Fig 3A; Table 1). As weapons grow large, mechanical advantage (and therefore weapon force output) is predicted to decrease in the absence of compensation and limit the relationship between weapon size and weapon force output(Eq 1; [29,38]In frog-legged beetles, however, mechanical advantage was maintained across all animals and absolute force production increased with weapon size (Figs 3A and 5; Table 1). This suggests frog-legged beetles employ one or more compensatory mechanism, which partially mitigates the mechanical limits predicted to hinder large weapon sizes.

Here, we identified two potential compensatory mechanisms, proportional growth of weapon/hindleg lever components and disproportionate growth of femur muscle mass. Overall, male frog-legged beetles do not experience mechanical disadvantage as weapons grow large, since they compensate for the increase in output lever length associated with increased in weapon size by similarly increasing input lever length. Male frog-legged beetles display longer input *and* output levers than females, which result in constant mechanical advantage across weapon sizes and between sexes (Fig 5; Table 1; S1 Table).

In addition, in male frog-legged beetles, femur muscle mass (F_in_) increased hyperallometrically with body size (*β* > 3; Fig 6A; Table 1), which is consistent with compensatory mechanisms identified in other systems [38]. It should be noted, however, that both absolute *and* relative weapon force output should increase with weapon size, given disproportionate muscle growth and the observed maintenance of mechanical advantage (Fig 5; Table 1). Clearly, there are as-yet undiscovered limits to weapon force production in this system (mechanical and/or behavioral), and further work is necessary to uncover why exactly weapon force output scales hypoallometrically in the frog-legged beetle.

Male leaf-footed cactus bugs showed no significant relationship between weapon force output (F_out_) and weapon size (Fig 3B; Table 1). This result was surprising given the observed increase in mechanical advantage with weapon size (Fig 5; Table 1). However, leg muscles remained proportional across all weapon sizes (Fig 6B; Table 1), which may explain why weapon force output did not increase with weapon size in males of this species. This result was unexpected given the established role hindleg weapons play in male-male competition [26,63], and the maintenance of mechanical advantage across weapon sizes. One explanation for this trend is that these hindlegs may be under relatively weak selection for increased force production in the context male-male combat. Instead, the hindlegs of leaf-footed cactus bugs may serve a greater role as intersexual signals of male quality, a behavioral context in which weapon force output is not an important component of fitness and hindlimb area, rather than force production, is under strong selection for increased size. Indeed, previous work suggests hindleg area is an honest indicator of overall quality [65–68] and recent studies have detected directional selection for increased hindleg area in the wild [63]. If true, then focal animals may have been unwilling to perform at full capacity during squeezing trails, since their hindlegs function primarily as display signals rather than weapons.

Alternatively, the ability to squeeze an opponent between both femurs, rather than between the femur and tibia of a single leg (as measured here), may be the most relevant metric of fighting success in this system (personal observation; Miller lab, University of Florida). Either scenario would result in an underestimation of weapon force output and could explain the observed non-significant relationship between weapon size and weapon force output. While we maintain our measures of weapon size, L_in_, and L_out_, are relevant in this system and to understanding the forces produced by these weapons, further investigation is necessary to establish exactly how weapon length and force production influence the outcome of male-male competition in the leaf-footed cactus bug, and what role, if any, these traits play in overall reproductive success.

### Compensatory muscle growth and honest signaling in the frog-legged beetle

Sexually selected weapons act as signals of quality and weapons of male-male battle. In both contexts, honesty is essential. Weapon size must honestly display quality to potential mates [18–20] and fighting ability to rival males [19,21–24,26,48] and, when tested in combat by similarly armed opponents, large weapons must produce sufficient force [69]. If not, receivers are predicted to focus to other, more reliable indicators of quality/fighting ability and selection for large weapons/signals should relax. Honesty in sexually selected weapons can be maintained through several mechanisms, including exquisite sensitivity to stress [74], parasite load [75,76], environmental condition [77], and intrinsic cost associated large structures [78,79]. The latter is particularly relevant to weapon systems where large, conspicuous structures often hinder the animals that bear them [80–85]. When present, the costs of sexually selected weapons typically increase with trait size, so only the largest animals can develop and wield large weapons and high quality signals are restricted to high quality males [28,78,79,86].

We suggest the compensatory muscle growth identified in frog-legged beetles comes at a cost and, through that cost, functions as mechanism of honesty. Muscle is notoriously expensive to develop [87–89] and maintain [80,82,90–94]. In preserving absolute weapon force output through compensatory muscle growth, frog-legged beetles may experience added metabolic [80,94] and locomotor [80,82] strain. For example, fiddler crabs with large, muscular claws suffer from disproportionally high resting metabolic rates [80,94], while stag beetles with large mandibles experience decreased flight performance resulting from their heavy, muscular jaws [82]. Such costs are consistent with theoretical models of handicap and indicator traits, where cost helps maintain the honesty/integrity of sexually selected traits as signals [78,79,86,95–98]. We therefore suggest that compensation for mechanical disadvantage through muscle growth may contribute to the integrity of weapon size as an honest indicator of quality and fighting ability in this system.

## Conclusion

The size of sexually selected weapons is critical to their role as honest signals. Weapons signal overall quality to potential mates and display fighting prowess to rival males. In both contexts, large traits are favored. However, selection for large, conspicuous signals is likely balanced by the need for weapons to perform well during combat. Here, we analyzed the relationship between weapon size and weapon force production (i.e., performance) in two systems, frog-legged beetles (*S*. *femorata*) and leaf-footed cactus bugs (*N*. *femorata*). In male frog-legged beetles, weapon force output scaled hypoallometrically with weapon size. This is partially consistent with lever theory, where both absolute and relative force output are predicted to decrease as weapons become large [29,38]. However, absolute force output appears to be maintained in this system through the maintenance of mechanical advantage across all weapon sizes and a disproportionally steep scaling relationship between leg muscle mass and body size. Alternatively, male leaf-footed cactus bugs showed no relationship between weapon size and force production, potentially reflecting the importance of hindleg area as an intersexual display of male quality rather than a force-producing weapon of male-male competition.

Overall, we suggest that when weapon force production is an important component of reproductive success, and animals experience mechanical limits to weapon force production, the evolution of compensatory mechanisms is likely (reviewed in [99]). We also suggest that some compensatory mechanisms, such as muscle growth in the frog-legged beetle, could enhance signal honesty in the context of sexual selection, both by disproportionately increasing metabolic or other costs associated with the largest male weapons and by maintaining fight performance at even the largest weapon sizes. Clearly, more work is required to understand the realized cost of heavily muscled weapons, how this influences individual fitness in the wild, and the ubiquity of the trends described here.

## Supporting information

S1 Table Summary of morphological measurements. (DOCX)

S1 Fig Schematic of constructed force transducer. A) Rigid metal bar used to stabilize the transducer stationary during trials. B) Flexible, brass arms that bend during squeezing trials. C) Needles that the animals squeeze during trials. Squeezing force (red) causes deformation in brass arms (B). Deformation is recorded by strain gauges (blue) in a full bridge configuration, as they are placed under tension (T1 and T2) and compression (C1 and C2).
(TIFF)

S2 Fig Lever components of A) *Sagra femorata* and B) *Narnia femorata* hindlimbs. (TIFF)
