# Supplementary material for: Overcoming mechanical adversity in extreme hindleg weapons
Source: PLoS One. 2018 Nov 7;13(11):e0206997. doi: 10.1371/journal.pone.0206997 (PMC6221328; doi:10.1371/journal.pone.0206997)
Supplement: S1 Table — (DOCX) [file pone.0206997.s001.docx]

| **Species** | **Sex** | **Trait** | **Min** | **Max** | **Mean** | **SE** |
| --- | --- | --- | --- | --- | --- | --- |
| *S. femorata* | male | Weapon size (femur length) | 13 mm | 6.1 mm | 9.84 mm | 0.123 |
| *S. femorata* | female | Weapon size (femur length) | 5 mm | 8.3 mm | 7.191 mm | 0.058 |
| *S. femorata* | male | Body size (elytra length) | 9.7 mm | 15.8 mm | 13.569 mm | 0.128 |
| *S. femorata* | female | Body size (elytra length) | 10.5 mm | 14.3 mm | 12.82 mm | 0.906 |
| *S. femorata* | male | Input lever (L_in_) | 0.251 mm | 0.852 mm | 0.7 mm | 0.012 |
| *S. femorata* | female | Input lever (L_in_) | 0.378 mm | 0.733 mm | 0.505 mm | 0.005 |
| *S. femorata* | male | Output lever (L_out_) | 2.918 mm | 9.780 mm | 8.053 mm | 0.121 |
| *S. femorata* | female | Output lever (L_out_) | 4.363 mm | 8.426 mm | 5.817 mm | 0.055 |
| *N. femorata* | male | Weapon size (femur area) | 3.742 mm | 8.55 mm | 5.677 mm | 0.160 |
| *N. femorata* | female | Weapon size (femur area) | 3.912 mm | 7.556 mm | 5.418 mm | 0.133 |
| *N. femorata* | male | Body size (body length) | 9.2 mm | 12.021 mm | 10.312 mm | 0.094 |
| *N. femorata* | female | Body size (body length) | 10.1 mm | 13.28 mm | 11.54 mm | 0.121 |
| *N. femorata* | male | Input lever (L_in_) | 0.278 mm | 0.475 mm | 0.354 mm | 0.006 |
| *N. femorata* | female | Input lever (L_in_) | 0.246 mm | 0.426 mm | 0.348 mm | 0.007 |
| *N. femorata* | male | Output lever (L_out_) | 3.962 mm | 4.937 mm | 4.407mm | 0.038 |
| *N. femorata* | female | Output lever (L_out_) | 3.178 mm | 5.079 mm | 4.410 mm | 0.06 |
