# Supplementary figures and images for: Overcoming mechanical adversity in extreme hindleg weapons
Source: PLoS One. 2018 Nov 7;13(11):e0206997. doi: 10.1371/journal.pone.0206997 (PMC6221328; doi:10.1371/journal.pone.0206997)

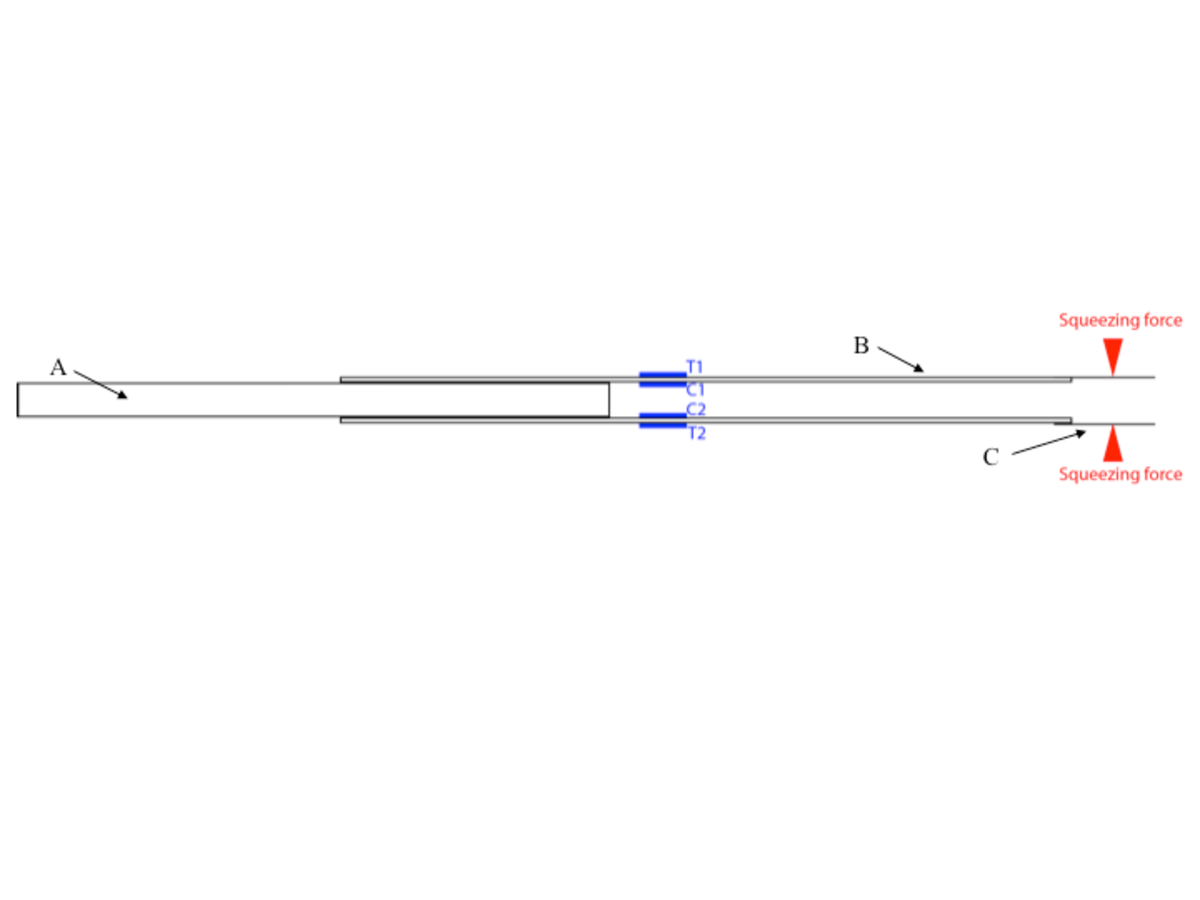

Supplement: S1 Fig — A) Rigid metal bar used to stabilize the transducer stationary during trials. B) Flexible, brass arms that bend during squeezing trials. C) Needles that the animals squeeze during trials. Squeezing force (red) causes deformation in brass arms (B). Deformation is recorded by strain gauges (blue) in a full bridge configuration, as they are placed under tension (T1 and T2) and compression (C1 and C2). (TIFF) [file pone.0206997.s002.tiff]

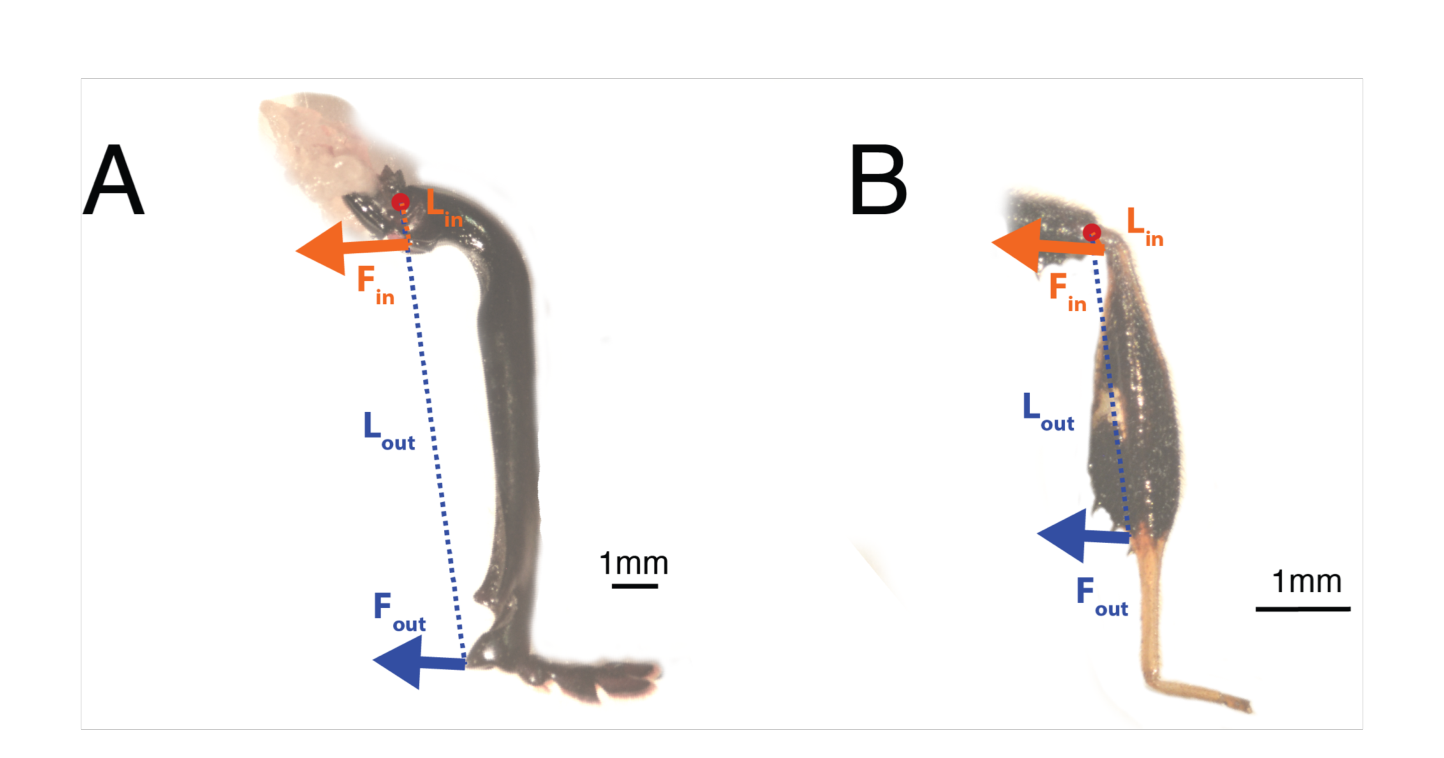

Supplement: S2 Fig — (TIFF) [file pone.0206997.s003.tiff]
